# Symptoms burden and rehabilitation preference after an episode of COVID-19: A patients survey

**DOI:** 10.1177/14799731231177316

**Published:** 2023-05-16

**Authors:** Munyra Alhotye, Enya Daynes, Charlotte Gerlis, Sally J Singh

**Affiliations:** 1 Department of Respiratory Sciences, 4488 University of Leicester, Leicester, UK; 2 Department of Respiratory Therapy, King Saud Bin Abdulaziz University for Health Sciences, Riyadh, Saudi Arabia; 3 Centre for Exercise and Rehabilitation Science (CERS), 573775 University Hospitals of Leicester NHS Trust, Leicester, UK

**Keywords:** COVID-19, COVID-19 symptoms, long COVID, rehabilitation

## Abstract

**Background:**

After COVID-19 infection, individuals can experience a variety of symptoms that might require further treatment. Early data showed the value of adapted pulmonary rehabilitation programmes and technology-based interventions. To develop appropriate services, it is important to understand the symptom burden and the preferred mode of rehabilitation delivery.

**Methods:**

Post-hospital discharge (H) and post-community-managed (C) individuals received a follow-up call. A survey was completed to assess the most burdensome symptoms for which the patients would require support and their preference for the mode of rehabilitation delivery.

**Results:**

Overall, 160 individuals who received a follow-up call completed the survey (51.2% male, mean [SD] age 54 [15] years) and 126 (78.8%) were post-hospital, while 34 (21.3%) had community-managed infections. A total of 101 (63.1%) reported that COVID-19-related symptoms were affecting their daily activities, and 106 (66.3%) reported their desire to be more active. The most common symptoms identified as needing support were fatigue and shortness of breath. Both groups expressed a preference for a face-to-face group programme (C: 54.8%; H: 46.8%), while (38.7%) of post-community-managed individuals and (40.3%) post-hospital patients preferred a supported digital rehabilitation programme. Few opted a non-digital home-based programme (C: 3.2%; H:12.9%, respectively).

**Conclusion:**

The survey responses indicated a significant symptom burden that may benefit from an intervention such as rehabilitation. Preferences for rehabilitation indicated that a face-to-face intervention was preferred by the majority, with a large proportion preferring digital intervention.

## Introduction

Coronavirus disease 2019 (COVID-19), caused by severe acute respiratory syndrome (SARS-CoV-2), was declared as a worldwide pandemic by the World Health Organisation (WHO).^1^ Individuals infected with SARS-CoV-2 might experience a variety of ongoing symptoms such as fatigue, cough, shortness of breath, dizziness, palpitations, increased levels of anxiety, and depression.^2,3^ The National Institute for Health and Care Excellence (NICE) guidelines classified the signs and symptoms of COVID-19 that lasted more than 12 weeks after the initial onset of an infection as “long COVID” or “post-COVID-19 syndrome”.^4,5^ It became obvious that a recovery intervention would be required to improve physical and psychological functions and enable COVID-19 patients to continue with their lives as normal.^6^ A clinical task force published by the European Respiratory Society suggested the need for a comprehensive rehabilitation programme after recovery.^7^ It has been reported that a pulmonary rehabilitation programme (PR) could be adapted to meet the needs of individuals with COVID-19.^8^ A PR programme is a complex intervention that involves education, assessment, physical training, and psychological support to improve the quality of daily life of individuals with chronic respiratory conditions.^9^ Symptoms presented in those recovering from COVID-19 such as dyspnoea, fatigue, cough, reduced exercise capacity, and increased levels of anxiety are similar to those with chronic respiratory diseases who benefit from rehabilitation.^10^ Rehabilitation programmes have been developed and tailored to accommodate individuals following an infection of SARS-CoV-2, and have reported improvements in respiratory symptoms, fatigue, and exercise capacity in both technology-based and traditional face-to-face programmes.^11,12^ Moreover, an improvement in quality of life, anxiety, and depression scores was reported by those who were discharged from hospital, and the programme was acceptable to participants with a high retention rate, and no adverse events were reported during enrolment.^12^

In this study, we aimed to understand the most bothersome symptoms individuals required support with and their preference for the mode of delivery following COVID-19 infection. To help develop an appropriate service, it is important to assess individuals’ symptoms and their preferred rehabilitation services.

## Methods

This cross-sectional survey for individuals with post-COVID-19 symptoms was conducted at the Biomedical Research Centre (BRC), University Hospitals of Leicester; and gained an ethical approval from the National Health Service Research Ethics Committee (Reference 17/EM/0156). Approximately 3 months after discharge, those who were discharged (H) from Glenfield Hospital, Leicester were automatically followed up, and individuals with community-managed (C) infections who were referred to the hospital for reported symptoms only received a follow-up call. Individuals were approached consecutively, and the survey was administered by a highly specialised physiotherapist (ED). All study participants were aged ≥18 years. Those unable to communicate fluently in English were excluded. The survey was designed based on a previous survey formulated by the National Institute for Health Research (NIHR) Global RECHARGE Group.^13^ This survey consisted of ten closed questions and free text for additional comments, and was completed between February and October 2021. (Supplement 1).

The first part of the questionnaire focused on the participants’ basic demographic information, including age, gender, their current activity level, and if they like to be active. They were also asked about the most bothersome symptoms that they need assistance with, and if they were interested in participating in a COVID-19 rehabilitation programme. In addition, they were asked about their preferences for the delivery of the rehabilitation programme, and they had to choose one option. The approximate time to complete the survey was 5–10 min. The results of the survey would be used to assist in the design of a flexible approach to rehabilitation interventions.

After completing the survey, the data were then transferred to the statistical package for the social science (SPSS) V.25 for further analysis.^14^ Simple descriptive statistical analysis was used, and the data were presented as frequency, percentages and mean standard deviation as appropriate.

## Results

Overall, 160 individuals who were approached, completed the survey (51.2% male, mean [SD] age 54 [15] years); 126 (78.8%) were patients who had been discharged from hospital, while 34 (21.3%) had community-managed infections. At the time of this study, the predominant COVID-19 strain in the UK was Delta variant.^15^

A total of 101 (63.1%) reported that COVID-19-related symptoms were affecting their daily activities, and 106 (66.3%) reported their desire to be more active.

**Figure 1.**
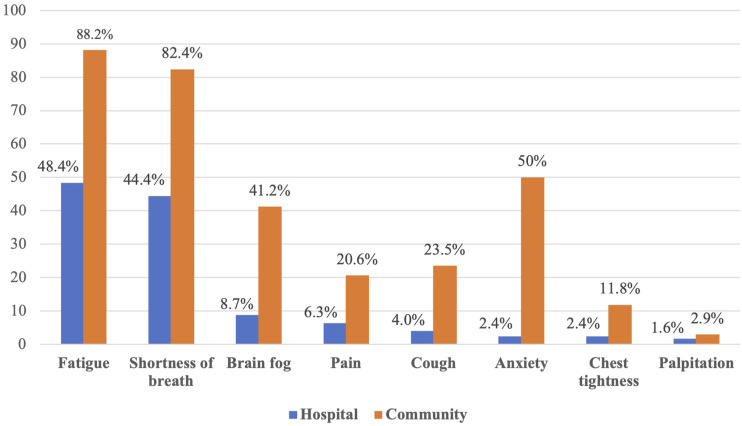
Reported bothersome symptoms.

The most common symptoms reported among participants from each group were fatigue (C: 82.4%; H: 44.4%) and shortness of breath (C: 88.2%; H: 48.4%). Higher anxiety levels were reported by half of the community-managed participants compared to very few in the hospital discharge group (C: 50%; H: 2.4%). Other important symptoms were brain fog (C: 41%; H: 8.7%), cough (C: 23.5%; H: 4%), and pain (C: 20.6%; H: 6.3%). Chest tightness (C: 11.8%; H: 2.4) and palpitations (C: 2.9%; H: 1.6%) were less frequently reported as troublesome symptoms (Figure 1).

The majority of community-managed participants (31; 91%) and (63; 50%) post-hospital reported their willingness to participate in a programme that would help to reduce their post COVID symptoms and improve their physical performance and quality of life. Of these, the dominant symptoms were fatigue (C: 87.2%; H: 66.6%) and shortness of breath (C: 74.1%; H: 63.4%).

**Figure 2.**
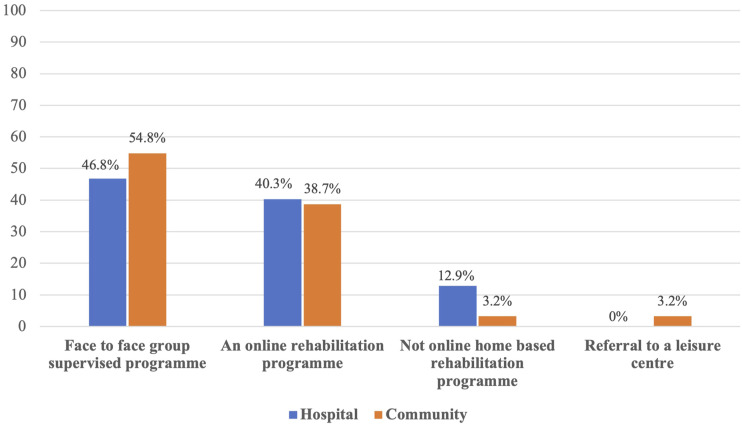
Preferred rehabilitation programme.

The preferred mode of programme delivery was also assessed, and both groups expressed a preference for a face-to-face supervised group programme (C: 54.8%; H: 46.8%), while 38.7% of participants who had had a community-managed infection and 40.3% of participants who had been discharged from hospital preferred a digital rehabilitation programme supported by a healthcare professionals. Only a few expressed a desire for a non-digital home-based programme (C: 3.2%; H: 12.9%). A small number of individuals who had had a community-managed infection expressed a preference for a programme based in a leisure centre (C: 3.2%) (Figure 2).

## Discussion

In this study, we investigated the ongoing and important symptoms of COVID-19 (lasting more than 3 months after the initial onset of infection) in individuals who had been discharged from hospital and those who had community-managed infections. Based on our results, shortness of breath and fatigue appeared to be the most bothersome symptoms in both groups. In addition, anxiety and brain fog were reported more frequently by community-managed participants than those who were discharged from hospital. Other symptoms such as coughing and pain were reported equally by both groups, while fewer participants reported signs of chest tightness and palpitations.

We observed a higher percentage of reported symptoms in the community-managed group. It should be noted that we only surveyed those who presented with symptoms and were referred to the management services from the community, while we surveyed all those who were discharged from hospital. The symptoms reported in our study were similar to those reported in previous studies that have identified fatigue, shortness of breath, and generalised pain as the most prevalent symptoms documented in individuals post-COVID-19.^2,16^ Furthermore, our study is consistent with a previous review conducted by Aiyegbusi et al., who investigated the most prevalent symptoms reported by individuals post-COVID-19. They found that fatigue (47%) and shortness of breath (32%) were the most prevalent symptoms in individuals who had been hospitalised.^17^ These symptoms were also reported by those who were not hospitalised.^18^

Importantly, our survey assessed the most bothersome symptoms rather than the most prevalent symptoms in post-hospital discharge and community-managed settings, and specifically sought to confirm the most important symptoms to inform service development. We are aware of the symptoms reported in the literature, but as healthcare professionals, we need to gauge the most troublesome symptoms to develop rehabilitation services that can address multiple symptoms at any one time. A few reports have highlighted the need for a rehabilitation programme and identified that pulmonary rehabilitation (PR) could be adapted to accommodate those with long COVID symptoms.^7,8,19^ In our study, we found that the majority of participants in both groups were enthusiastic about participating in a programme that would help them to manage their symptoms and improve their physical activity levels and quality of life. In the development of any rehabilitation intervention as our data indicated that shortness of breath, fatigue and anxiety were the most burdensome symptoms and healthcare professionals need to be equipped with knowledge and skills to offer support to the post COVID-19 community. Staff delivering PR have a considerable experience in managing dyspnea and anxiety, but may be familiar with fatigue management, and the impact of post exertional symptoms exacerbation.^20^

Due to the burden of long COVID, we anticipated that there would be a significant demand for rehabilitation services. Therefore, it would be logical to devise flexible ways of delivering the rehabilitation services, and an option for face-to-face and digital interventions may help meet the demand and offer patients a choice of interventions, as indicated in this data. This study assessed individuals’ preferences for rehabilitation programme delivery when it comes to the management of post-COVID-19 symptoms. They had a choice between a face-to-face programme in a group supervised by a team of healthcare professionals or a programme manual to be used at home with support from healthcare professionals. The delivery options included an online-based comprehensive programme and a leisure centre scheme. The majority of participants in both groups preferred a supervised face-to-face rehabilitation programme followed by an online-based programme. A small number of participants opted for the home-based programme manual, while a couple of community-managed participants preferred the leisure centre option.

The study has several limitations. Firstly, the study was not asked individuals about their digital competency. However, the appetite for technology based programmes was much higher than what we have observed in COPD population.^21^ The COPD survey didn’t evaluate digital competency, but the average age of our study population was about 20 years younger than COPD population surveyed. Another limitation that patients’ comorbidities and length of hospital stay were not recorded. However, the study sought to gain an initial insight about the symptoms burden and thoughts about enrolling into rehabilitation programme.

This study demonstrated the need for flexible rehabilitation options to alleviate the persistent symptoms of COVID-19 infection in those who have been discharged from hospital or those whose infections have been community managed. This will require the delivery of a range of options to meet patients’ preferences and demands.

## Conclusion

The survey responses indicated a significant symptom burden that may benefit from an intervention such as rehabilitation. Preferences for rehabilitation indicated that a face-to-face intervention was preferred by the majority, with a large proportion preferring digital intervention.

## Supplemental Material

Supplemental Material - Symptoms burden and rehabilitation preference after an episode of COVID-19: A patients survey Click here for additional data file. Supplemental Material for The formal rationality of artificial intelligence-based algorithms and the problem of bias by Munyra Alhotye, Enya Daynes, Charlotte Gerlis and Sally J Singh in Chronic Respiratory Disease
