# Supplementary material for: Symptoms burden and rehabilitation preference after an episode of COVID-19: A patients survey
Source: Chron Respir Dis. 2023 May 16;20:14799731231177316. doi: 10.1177/14799731231177316 (PMC10191828; doi:10.1177/14799731231177316)
Supplement: Supplemental Material - Symptoms burden and rehabilitation preference after an episode of COVID-19: A patients survey [file sj-pdf-1-crd-10.1177_14799731231177316.pdf]

Having COVID may have left you with a number of symptoms that are bothering you. We are developing recovery services for people like you and would welcome your opinion to help shape our services. We envisage this service will address important aspects of your physical and mental health.
We would be grateful if you could take a minute to complete this brief questionnaire on your views.

Please circle:

1. Male / Female

2. Age Group:

Under 40 / 40-50 / 50-60 / 60-70 / 70-80 / over 80

3. Have your symptoms stop you doing any daily activities?

Yes / No

4. Does your current level of activity bother you?

Yes / No

5. How active do you think you are compared to your pre COVID levels?

Please mark a cross on the scale below which runs from 0 (as far away as I could imagine) to 10 (back to normal)

|---|---|
0 1 2 3 4 5 6 7 8 9 10

6. Would you like to be more active, less breathless and/or less tired when you do daily activities?

Yes / No

7. What is/are your most troublesome symptom(s) that you would like support
…………………………………………………
…………………………………………………
…………………………………………………
…………………………………………………

PLEASE TURN OVER

8. Would you be interested in participating in a COVID recovery programme?

- If Yes….Please go to Question **8b.**
- If No… Please select option:

† No, generally improving

† Other: please provide a reason below:

… …

… …

8b. If yes, where would you prefer to do this programme?

**Please tick only ONE**		**TICK**
1	An online comprehensive home based programme supported by health care professionals	
2	A comprehensive home based programme (not online based for example a work book) supported by health care professionals	
3	Face to face in a group supervised by a team of healthcare professional	
4	I would consider referral to a leisure centre scheme	

9. Do you have access to a computer/tablet?

Yes / N

10. Do you use a mobile phone?

Yes / No

10b. If yes, is it a 'smart' phone?

Yes / No

Thank you for taking the time to complete this questionnaire.
